# Bone mineral density assessment by DXA in disease-associated osteoporosis: the limitations

**DOI:** 10.3389/fendo.2026.1768509

**Published:** 2026-03-09

**Authors:** Kamyar Asadipooya, Loren Wissner Greene

**Affiliations:** 1 Division of Endocrinology and Molecular Medicine, Department of Medicine, University of Kentucky, Lexington, KY, United States; 2 Holman Division of Endocrinology, Department of Medicine, NYU Grossman School of Medicine, New York, NY, United States

**Keywords:** BMD, DXA, FRAX, HR-pQCT, TBS

## Abstract

Measuring bone density by DXA is a routine means of predicting fracture risk. These 2–dimensional images have limitations and may underestimate the risk of fracture due to inadequate evaluation of bone strength. The inclusion of clinical risk factors, calculating FRAX and adding TBS values all improve our ability in bone screening by DXA. However, secondary osteoporosis is not always associated with classical risk factors for bone loss. In addition, unrecognized underlying conditions may contribute to further bone loss and increased risk of low trauma fracture. Therefore, new technologies that assess bone microarchitecture, such as HR-pQCT, can improve our risk assessment for fragility fracture.

## Introduction – limitations of bone density

Bone loss and microarchitectural deterioration after menopause or secondary to disease conditions leads to increased risk of fragility fracture. The current and usual method to assess fracture risk is bone mineral density (BMD) measurement by dual energy X-ray absorptiometry (DXA). DXA estimates BMD by measuring attenuation of X-ray beams comparing attenuation at different energies. As DXA scan uses 2-dimensional images to demonstrate actual 3-dimensional objects, there are obvious technical limitations on these measurements as it employs “areal” or 2D bone density to predict 10-year fracture risk (1). In addition, fractures may result from bone overload and failed defense mechanisms that are mainly secondary to other parameters including impaired bone strength, decreased fracture toughness and fatigue stress, not measured in standard DXA. The fracture toughness is a bone material property other than strength to resist cracking. Impaired fatigue stress, also called fracture strength or fatigue fracture, is the result of repeated cycles of small loads that overwhelm the bone strength. Consequently, fracture risk assessment might be improved by assessing load and detecting impaired bone strength, fracture toughness and fatigue strength.

Currently, DXA BMD is the recommended approach for diagnosing osteoporosis by the World Health Organization (WHO) and the International Society of Clinical Densitometry (ISCD). According to the WHO classification normal BMD is T-score ≥ -1.0; osteopenia is T-score < -1.0 and > -2.5; and osteoporosis is T-score ≤ -2.5. However, the sensitivity of DXA BMD for fracture is low and, by population size, most individuals with history of low trauma fracture have had normal BMD or osteopenia (1). The DXA limitations could be due to artifact errors, the type of measurement, which measures areal BMD, and not volumetric BMD as mentioned above and these measurements cannot assess a more detailed measurements of bone structure and strength. Furthermore, in the situations in which fragility fractures occur, those inaccuracies increase in situations of impaired bone microarchitecture and tissue quality.

However, since the measurement of load is almost never available in a preventive approach to reduce fracture risk, we should also try to focus on the evaluation of the resistance ability of bone against mechanical loads, as determined by bone density, morphology, cortical thickness, trabecular structure, geometry and mechanical properties. As a result, a proper assessment of fragility fracture requires investigating not only bone mineral density but also texture and size, material properties, material distribution, quality, microarchitecture and geometry of the bone (2, 3).

In this review, we will discuss the advantages and limitations of DXA BMD for evaluating the fracture risk in secondary osteoporosis. We hope to highlight the conditions in which there may be an inaccurate assessment of risk of fracture based on bone density. It is also important to identify the risk factors of fragility fracture, which might guide us to use an alternative approach such as trabecular bone score (TBS), quantitative CT or ultrasound, high resolution CT or MRI, finite element analysis (FEA), reference point indentation (RPI), bone biopsy and histopathology for predicting fracture.

## Diabetes and fracture risk assessment

Evidently, diabetes is associated with increased risk of hip and non-vertebral fracture. There are several proposed mechanisms including an increased risk of fall itself especially with rapid changes in glucose levels or actual hypoglycemia, balance problems due to peripheral neuropathy and visual impairment. There are also microvascular damages, impaired bone metabolism, and bone material damage mainly due to advanced glycation end products (4, 5). DXA is a widely available test for assessing fracture risk. Patients with T1DM often have decreased BMD but the amount of decrease in BMD often underestimates the fracture risk. In T2DM patients, BMD often is increased, yet there may be a contradictory increase in fracture risk (4, 6). This information highlights the limitations of DXA in predicting fracture risk in diabetic patients. The bone loss and microarchitecture damages associated with diabetes make microarchitecture studies and volumetric BMD measurements superior to DXA scan for predicting fracture. In addition, TBS (7) and peripheral quantitative computed tomography (pQCT) at the tibia and pQCT based finite element analysis (pQCT-FEA) (8) can predict fracture risk in diabetic patients better than DXA. TBS is a method of estimating trabecular microarchitecture and FEA is a computational method to assess bone strength. The typical model for predicting fracture risk is the Fracture Risk Assessment Tool (FRAX). However, TBS can be a BMD-independent predictor of fracture in people with or without diabetes (7). The novel tools such as bone phenotype (low density, low volume, and healthy bone phenotypes) and Microarchitecture Fracture Risk Assessment Calculator (μFRAC) may improve prediction of osteoporotic fracture, using HR-pQCT parameters, clinical risk factors (sex, age, height, weight, and history of previous fracture) and femoral neck areal BMD (9, 10), in patients with diabetes. While invasive methods such as micro indentation and bone histomorphometry may also be useful, they are time-consuming with limited applicability for clinical practice (11, 12).

## Chronic kidney disease and fragility fracture

Chronic kidney disease (CKD) is associated with higher risk of fragility fracture and the risk increases with progression of kidney dysfunction. The term CKD mineral and bone disorder (CKD-MBD) or renal osteodystrophy (ROD) is used to explain a clinical syndrome that is associated with fractures, extra skeletal calcification, and cardiovascular events. Disturbances in bone and mineral metabolism occur in early stage CKD and increase in almost all patients with advanced CKD (13, 14). ROD is caused by abnormalities of mineral including calcium, magnesium and phosphate, vitamins (including active vitamin D synthesis), hormones (mainly PTH), leading to bone metabolism disorders that affect quantity, quality and remodeling process of the bone. The proper assessment of bone quantity and quality in ROD can help predict the risk of fracture. However, assessment of bone quantity by DXA in CKD has limitations, as it does not measure volumetric BMD and bone quality parameters. In addition, the management of ROD requires information about bone loss, microarchitecture changes, stage and progression of CKD and differentiation between the etiologies, including high bone turnover versus low bone turnover. As a result, information about PTH and bone turnover, and application of tools such as TBS, HR-pQCT-FEA and sometimes a bone biopsy for histopathology assessment may be necessary for diagnosis and treatment (15–18).

## Predicting fragility fracture in endocrine disorders

Primary hyperparathyroidism (PHPT) mainly affects cortical bone more than trabecular bone and is associated with increased risk of fracture at the vertebrae and forearm (19). There are controversies about the ability of BMD to predict fracture in PHPT patients. Vignali reported lower BMD at all sites in women with PHPT and history of fracture (20), while Kaji showed lower BMD at the lumbar spine, but not the femoral neck and radius, in female patients with PHPT and fracture (21). A case-control study from Denmark reported that men and women with PHPT and fracture had significantly lower BMD at the total hip and forearm but not the lumbar spine, which may indicate that BMD measurement by DXA may predict the fracture risk at the hip and forearm, while it is not helpful for vertebral fracture assessment (22). TBS and TBS-adjusted FRAX may improve fracture risk prediction (23–29). However, there are controversies around the sensitivity of TBS to predict vertebral fracture (29–31). HR-pQCT of distal radius and tibia can also provide a better assessment of bone health, including geometry, volumetric density and microarchitecture, in PHPT (32–37).

Hypoparathyroidism is an uncommon endocrine disorder, which leads to the reduction of bone remodeling due to lack of parathyroid hormone (PTH). Overall, the impact of chronic hypoparathyroidism on fracture risk, especially vertebral fracture, is unclear. It is been reported that hypoparathyroidism may (38–41) or may not increase fracture rate (42–47). A meta-analysis by Pal in 2021 reported that increased risk of vertebral fractures was seen only in nonsurgical hypoparathyroidism (48). Hypoparathyroid patients typically have higher BMD values by DXA, even with transient postsurgical hypoparathyroidism for less than a year (49, 50). TBS is not helpful for predicting vertebral fracture as hypoparathyroidism subjects have comparable scores with controls, despite having higher BMD (38, 40, 43, 44). Other tools including HR-pQCT, FEA and bone material strength index (BMSi) measurement by impact microindentation at the anterior tibia can provide more information about bone quality and quantity, to improve fracture predictability (44, 50, 51).

Hyperthyroidism accelerates bone turnover, reduces bone mass and increases fracture risk. BMD values by DXA provide information to evaluate the bone strength and fracture risk in hyperthyroid patients. However, BMD does not demonstrate the effects of hyperthyroidism on bone remodeling and microarchitecture as precisely as HR-pQCT images. HR-pQCT of radius in hyperthyroid patients showed higher total area, and trabecular area, but lower cortical area, total volumetric bone mineral density (vBMD), cortical vBMD, cortical thickness, and estimated bone strength (52). Hypothyroidism lowers bone turnover, impairs bone remodeling, and leads to increased bone mineralization, but it does not affect BMD or fracture risk (53, 54). Generally, HR-pQCT would be a better option to assess bone health in the patient with thyroid disorders.

Meta-analysis of prospective cohorts’ reports that subclinical hyperthyroidism increases overall risk of hip, spine and non-spine fracture, especially in individuals 60 and older, but there is no similar correlation between fracture risk and subclinical hypothyroidism (55–58). In addition, DXA scan showed that subclinical hyperthyroidism was associated with greater annual bone loss at the femoral neck, but not at the lumbar spine. In contrast, there was no association between subclinical hypothyroidism and bone maintenance (59). TBS, HR-pQCT and histomorphometric studies provide more information about bone structure (60), but the differences between the patients with subclinical thyroid disorders and normal persons are minimal (61), so it is still considered to be cost effective to apply BMD by DXA for fracture prediction.

Glucocorticoids can affect bone cell activity and the remodeling process. Glucocorticoid replacement for adrenal insufficiency, primary or secondary, and as a treatment for inflammatory and pulmonary diseases often results in glucocorticoid-induced osteoporosis and attendant fragility fractures (62, 63). In addition, Cushing syndrome and even subclinical hypercortisolism are associated with bone loss, lower BMD and higher fracture risk, especially in the spine (62, 64). Despite the common use of DXA to predict fracture risk by assessing bone density and quality, patients with hypercortisolism also may suffer from unaddressed vitamin D and calcium deficiency, insulin resistance, suppression of growth hormone and sex steroids, muscle atrophy and secondary hyperparathyroidism, which all may play crucial roles to further increase fracture risk (62, 65). Therefore, DXA utility in predicting fracture in hypercortisolism patients is generally limited and TBS or HR-pQCT may better characterize the bone microarchitecture changes and estimate bone fragility (66, 67).

Growth hormone, either directly or indirectly through IGF-1, stimulates osteoblast proliferation and differentiation. Conversely, fragility fractures, especially vertebral fractures, are a recognized complication of acromegaly, mainly due to the increased bone turnover along with microarchitecture deterioration (68). Furthermore, prediction of bone fragility in acromegaly is a challenge as BMD can be normal or even increased, and biochemical remission of acromegaly does not always reduce fracture risk (68–70). However, lower lumbar spine TBS (70) and microstructure alterations assessed by HR-pQCT (71) seem promising in characterizing the bone microarchitecture changes to overcome limitations of DXA in acromegaly.

Growth hormone deficiency leads to lower bone mineral density and higher fracture rate. While recombinant human growth hormone replacement therapy increases BMD (72), its effect on fracture risk reduction is controversial (72–74). In addition, DXA has limitations for predicting fracture risk. HR-pQCT may provide more helpful information about microarchitecture and bone strength for fragility fracture prediction (75).

Male hypogonadism is associated with decreased BMD and increased fracture risk. Though BMD measurement by DXA provides accurate information regarding bone loss in hypogonadal men, HR-pQCT assesses microarchitectural changes that independently predict fracture risk. Testosterone treatment increases BMD mainly at the lumbar spine but clomiphene or anastrozole may reduce spine BMD. However, assessing bone microarchitecture by HR-pQCT could provide more information about the changes in cortical and trabecular bone with testosterone treatment (76–78).

Androgen deprivation therapy (ADT), a common treatment for advanced or recurrent prostatic cancer, is associated with loss of bone quality and density, with osteoporosis and ensuing increased risk of fracture (79–81). Additionally, maximum androgen blockade (combining androgen receptor pathway inhibitors with ADT) may further increase risk of fracture more than ADT alone (82). Although the current recommendation for monitoring bone loss and assessing fracture risk includes DXA and FRAX, DXA-based 3D modeling at the proximal femur and HR-pQCT at the distal tibia and radius would provide more information (83, 84). Generally, ADT is associated with reduced areal BMD, changes in volumetric BMD, and alteration of hip structural analysis assessed by 3D-DXA (83). Moreover, HR-pQCT at the distal radius and tibia can probably detect bone fragility better than either DXA and FRAX, by showing changes in microarchitecture and finite element analysis (stiffness and failure load), including bone mineral content, volumetric BMD, areal BMD, bone microarchitecture, bone strength, and body composition (85, 86). Lastly, there is no association between vertebral fracture and femoral neck areal BMD, but multivariate regression analysis shows that vertebral fracture was associated with impaired bone microarchitecture at the tibia, with a lower total and trabecular BMD, thinner cortex and trabeculae, higher trabecular area, and lower cortical area (84).

Aromatase inhibitors (AIs), the standard treatment for estrogen receptor-positive and hormone-positive breast cancers, are associated with deterioration in bone density and microarchitecture, in both cortical and trabecular components. Although, DXA detects these bone density changes, 3D-DXA may provide more data about bone damage (87). Furthermore, HR−pQCT is more sensitive in distinguishing the early changes in bone microarchitecture (88, 89).

## Hematologic disorders and bone loss

Hematologic disorders can have detrimental effects on bone health. This could be due to direct cellular effects on bone, catabolic effects of osteolytic mediators, or even from iron released from hemolyzed blood.

In multiple myeloma, the plasma cell infiltration and imbalance of bone remodeling decreased osteoblastogenesis, increased osteoclastogenesis, and osteocyte apoptosis, cause bone loss with lytic lesions. Moreover, the cytokines, such as receptor activator of NF-κ B ligand (RANKL), interleukins (IL-1 and IL-6), lead to osteoclastogenesis; while upregulation of mediators, such as dickkopf-1 (DKK1) and sclerostin, inhibit osteoblastogenesis (90). The quantitative methods of imaging by CT or MRI can improve the assessment of bone lesions in multiple myeloma patients (91).

In thalassemia, iron deposition can falsely increase X-ray attenuation values of trabecular bone, leading to a falsely elevated bone density reading. Therefore, quantitative computed tomography may underestimate the amount of bone loss in thalassemia patients, who are not sufficiently chelated (92).

Systemic mastocytosis (SM) is a hematologic disorder that can cause bone loss. It may lead to low bone mineral density (osteopenia or osteoporosis) and fractures. Generally, mast cell activation, proliferation, and infiltration into bone, as with other organs, are responsible for bone damage in SM. BMD measurement by DXA usually underestimates the risk of fracture (93). However, the fracture risk assessment can be improved by counting risk factors for fragility fracture, adding TBS values and applying HR-pQCT, especially for evaluating the bone microarchitecture and measuring volumetric BMD (94).

Of course, non-predictive high bone density occurs in vertebral fracture with bone collapse. DEXA may show inappropriately high bone density, not aligned with fracture risk, in such conditions as artefactual (degenerative diseases, osteoarthritis, ankylosing spondylitis, vascular calcification etc.) or true increase in BMD secondary to increased osteoblastic activity or osteoblastic tumor metastases (prostate cancer, mastocytosis, Paget’s disease, acromegaly, renal osteodystrophy, hepatitis C-associated osteosclerosis etc.) (95). Excess fluoride and strontium disproportionately elevate BMD measurements too (96, 97).

## Conclusion

**Figure 1:**
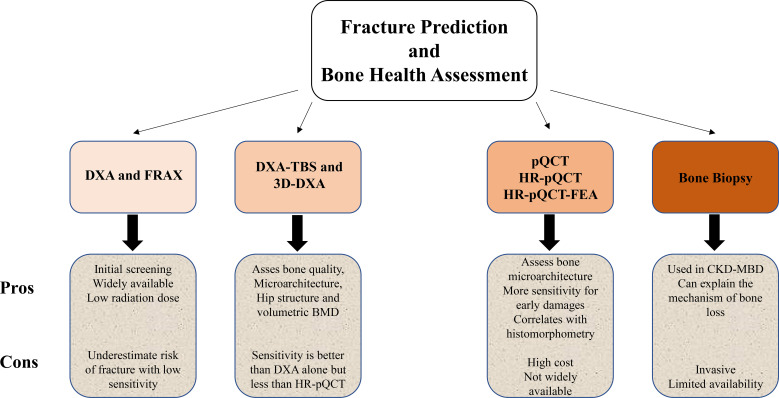
Flowchart graphic compares fracture prediction and bone health assessment methods: DXA and FRAX, DXA-TBS and 3D-DXA, pQCT/HR-pQCT/HRpQCT-FEA, and bone biopsy. Each method has pros and cons listed regarding sensitivity, availability, and invasiveness. DXA-FRAX, DXA-TBS are typically initial screening tools and use low dose radiation. 3D-DXA uses the same low dose radiation as a conventional standard 2D DXA. pQCT and HR-pQCT with or without Finite Element Analysis (FEA) also have comparable low radiation doses (98).

Proper diagnosis and prevention of fracture in secondary osteoporosis are more important than treatment of fracture. Applying a sensitive tool to predict fragility fracture is the cornerstone of screening patients with a history of underlying conditions that cause bone loss. These include considering risk factors, assessing bone quality and strength, and finally determining the degree of bone damage based on the severity of the primary medical condition. DXA-FRAX, DXA-TBS and even 3D-DXA are useful initial screening tools. However, applying a more sensitive method such as HR-pQCT is reasonable in high risk groups with multiple risk factors for fragility fracture (**Figure 1**). More investigations are required to introduce the best means of predicting fragility fracture, especially in various diseases that are associated with bone loss.
